# Medical thoracoscopic photodynamic therapy for metastatic pleural tumors with malignant effusion: an exploratory pilot study

**DOI:** 10.3389/fonc.2025.1634448

**Published:** 2025-09-26

**Authors:** Zhonglun Mai, Qianwen He, Yao Tang, Qixiao Feng, Ziqing Wu

**Affiliations:** ^1^ Department of Oncology, Cancer Center, Southern Medical University Hospital of Integrated Traditional Chinese and Western Medicine, Southern Medical University, Guangzhou, Guangdong, China; ^2^ Department of Geriatrics, Southern Medical University Hospital of Integrated Traditional Chinese and Western Medicine, Southern Medical University, Guangzhou, Guangdong, China; ^3^ Department of Pathology, Southern Medical University Hospital of Integrated Traditional Chinese and Western Medicine, Southern Medical University, Guangzhou, Guangdong, China; ^4^ Department of Respiratory and Critical Care Medicine, Jinshazhou Hospital of Guangzhou University of Chinese Medicine, Guangzhou, Guangdong, China

**Keywords:** medical thoracoscopy, photodynamic therapy, argon plasma coagulation, metastatic pleural tumor, malignant pleural effusion, non-small cell lung cancer

## Abstract

**Background:**

The optimal treatment for malignant pleural effusion (MPE) remains controversial. We previously explored the use of minimally invasive medical thoracoscopic thermal ablation [argon plasma coagulation (APC)] to reduce pleural tumors and manage effusion, achieving encouraging results. However, this method has some drawbacks.

**Methods:**

We conducted an exploratory pilot study to evaluate the efficacy of photodynamic therapy (PDT), alone or in combination with APC, in the treatment of pleural spread with MPE. Patients with plaque-like lesions received PDT alone with an energy density of 384 J/cm^2^. Nodular-like and mass-like lesions were treated with APC followed by PDT with energy densities of 480 and 576 J/cm^2^, respectively. The primary endpoints were the response and time to progression (TTP). The 1-year survival rate and safety were also assessed. Data from the PDT ± APC group were compared with those from the APC group from our previous study.

**Results:**

In total, 28 patients with non-small lung cancer (NSCLC) were enrolled. Eight patients underwent PDT alone and 20 patients underwent PDT and APC. At week 6, the overall response rate in the observation group was 82.1% (complete response in 15 cases and partial response in eight cases). At week 12, this rate had increased to 89.3%. The TTP was significantly longer in the PDT ± APC group than in the APC group (median, 20.7 vs. 14.2 months; *P* = 0.006; HR = 0.35). The 1-year survival rate was 75% (21/28).

**Conclusion:**

Medical thoracoscopic PDT ± APC therapy significantly improved TTP relative to APC alone and provided durable local effusion control among NSCLC patients with MPE.

## Introduction

Malignant pleural effusion (MPE) refers to pleural effusion caused by the metastasis of a primary malignant tumor of the pleura or a malignant tumor in another part of the pleura. Pleural metastasis typically appears as multiple masses of various sizes with a nodular appearance or as plaques (1). Lung cancer is the most common cause of MPE, accounting for almost half of all cases in some series (2). Up to 50% of patients with non-small cell lung cancer (NSCLC) develop MPE during the course of the disease (3). In the 8th edition of the American Joint Committee on Cancer’s TNM staging system for lung cancer, the presence of pleural seeding and MPE (M1a classification) leads to the designation of stage-IV disease (4). NSCLC with M1a metastasis has poor outcomes, with a median survival time of 11.5 months (5), especially for patients who cannot receive targeted therapy or immunotherapy.

The optimal treatment for MPE remains controversial. The most frequently applied therapeutic options for the management of pleural effusion, such as thoracentesis, pleural catheter placement, and pleurodesis, are mainly palliative, with no direct mechanical debulking effect on tumors in the pleura (6, 7). MPE therapy has improved continuously in recent years with the development and application of new techniques. In our previous study, we explored the use of medical thoracoscopic thermal ablation [argon plasma coagulation (APC)], a minimally invasive method that allows for more effective fluid drainage and the reduction of metastatic tumors (8). We found that APC therapy significantly improved the pleural effusion objective response rate (88.2% vs. 66.7%, *P* = 0.004) and time to progression (TTP; 13.7 vs. 7.3 months, *P* = 0.001) compared with catheter-based pleural drainage (8). Although these results are encouraging, APC therapy has some disadvantages. Endoscopic APC can be used to treat only macroscopic cancers limited to the parietal pleura; residual invisible and visceral pleural tumors remain untreated, resulting in a limited tumor reduction effect. In the effort to improve the curative effect of APC therapy, we sought to develop a new combined intrapleural therapeutic approach.

Photodynamic therapy (PDT) involves the use of photosensitive drugs combined with light irradiation to selectively destroy cancer cells (9). Recently, it has been used widely in cancer treatment; it is ideally suited for superficial tumors. Existing data on the use of PDT to treat malignant pleural tumors derive mainly from surgical cases (10, 11). Promising results, including the prolongation of survival times and achievement of good local control, suggest that PDT could be a beneficial alternative treatment for MPE (10, 11). However, more high-quality evidence is needed. Moreover, malignant pleural dissemination is generally considered to be contraindication to surgery, which, when performed, tends to be expensive and is often accompanied by complications. Thus, the popularization of surgery combined with intrapleural PDT in clinical practice is difficult.

Thoracoscopic PDT is being applied to pleural tumors with MPE at an increasing number of hospitals in China. However, very few clinical data on the efficacy of this technique have been published, and no consensus regarding the appropriate operative procedure has been reached. In light of these considerations, we conducted an exploratory pilot study with data from a prospective cohort receiving medical thoracoscopic intrapleural PDT, alone or combined with APC, to assess responses, TTPs, survival, and safety.

## Methods

### Study design and participants

This exploratory study was performed at the Southern Medical University Hospital of Integrated Traditional Chinese and Western Medicine, Guangzhou, China. Patients with NSCLC and malignant pleural disease (involving effusion and pleural lesions) were recruited between April 2019 and September 2022. All patients received systematic standard antitumor therapy in accordance with the National Comprehensive Cancer Network guidelines. All eligible patients underwent medical thoracoscopic intrapleural PDT, alone or in combination with APC therapy, according to the lesion type and size. Patients who underwent APC therapy in our previous study (8) were included for comparison. They were matched with patients recruited for the present study using propensity scores (1:1 ratio, caliper value = 0.2). The study was approved by the Ethics Committee of the Integrated Hospital of Traditional Chinese Medicine, Southern Medical University (no. NFZXYEC2019009), and was undertaken in accordance with the principles of the Declaration of Helsinki. Written informed consent was obtained from all patients before the performance of any study-specific procedure.

The inclusion criteria were: (1) primary NSCLC, (2) diagnosis of MPE based on imaging combined with pleural biopsy or fluid cytology, (3) symptomatic presentation, (4) Eastern Cooperative Oncology Group (ECOG) score ≤ 2, (5) concurrent receipt of standard systemic chemotherapy, and (6) estimated survival time > 3 months. Exclusion criteria were: (1) concurrent receipt of targeted therapy or immunotherapy; (2) obvious the lung trapping (<50% of lung surface in apposition to the chest wall), observed by computed tomography (CT); (3) intolerance of medical thoracoscopy; (4) photosensitizer allergy; (5) severe cardiopulmonary insufficiency and/or severe coagulation dysfunction; and (6) extensive pleural adhesions, detected by medical thoracoscopy.

### Procedures

A skin allergy test was administered before the photosensitizer injection. HiPorfin (3 mg/kg; Chongqing Maile Biopharmaceutical Co. Ltd.) was administered intravenously 40–48 hours before treatment and protected from light exposure for at least 1 month thereafter. Routine preoperative preparation, postoperative care, and conventional medical thoracoscopy were performed as described in our previous publication (8). After complete fluid aspiration and mechanical adhesion separation, intraoperative treatment was performed. All target therapeutic lesions were located in the parietal pleura.

#### PDT alone

PDT was applied to superficial and plaque-like lesions. A cylindrical laser fiber (1–4-cm length; Tianjin Guoyihuake Medical Technology Group Co. Ltd.) was introduced into the cavity and positioned against the central areas of parietal pleural lesions through the working channel of the thoracoscope. The cylindrical diffuser length was chosen according to the lesion size. The irradiated segments included margins of at least 0.5–1.0 cm beyond the edges of the targeted lesions. A diode laser system (Biolitec, Jena, Germany) was employed with a wavelength of 630 nm, power density of 800 mW/cm^2^, and total energy density of 384 J/cm^2^ for each treated area.

#### PDT combined with APC therapy

This procedure was applied to mass- and nodular-like lesions. APC was first employed to reduce the tumor burden as much as possible, followed by PDT. The specifics of the APC method are provided in our previous publication (8). Then, the treated areas were overlapped completely by PDT irradiation. The laser wavelength and power density were the same as used for PDT alone. The total energy density for each treated area was 480–576 J/cm^2^.

### Assessment

CT examinations were performed to monitor the pleural effusions at 6 weeks after initial treatment and every 6–8 weeks thereafter. The primary endpoints were the proportion of patients showing overall response [comprising complete response (CR) and partial response (PR)] and the TTP. The secondary endpoints were the 1-year survival rate and safety. The formula D2 × L was used on soft tissue windows setting of CT image to obtain a rough estimate of the pleural fluid volume (where D was the greatest depth of the effusion in the axial plane, and L was the greatest length of the effusion in the sagittal plane). The MPE estimation method is described in our previous publication (8). CR was defined as the absence of pleural effusion and the complete resolution of symptoms for >6 weeks, PR was defined as a >50% reduction in pleural effusion with the relief of symptoms lasting for >6 weeks, and NR was defined as the presence of pleural effusion larger than that defined by PR or the requirement for drainage within 6 weeks. The TTP was defined as the time from initial treatment to the first MPE recurrence, confirmed by CT imaging and requiring further ipsilateral pleural intervention.

### Statistical analysis

Categorical variables were compared using Pearson’s chi-squared test, and continuous variables were evaluated using the independent *t* test. CR, PR, and NR rates were analyzed using the Mann–Whitney *U* test. TTPs were calculated using the Kaplan–Meier method and compared using the log-rank test. A Cox regression model was used to estimate hazard ratios (HRs) with 95% confidence intervals (CIs). All statistical analyses were performed using the SPSS (version 26.0) and GraphPad Prism (version 5.0) software. *P* values < 0.05 were regarded as significant.

## Results

### Patients

Twenty-eight patients were enrolled as the observational group and received PDT ± APC therapy. Eight of these patients had plaque-like pleural lesions and PDT alone, and 20 patients had nodular or mass-like pleural lesions and received PDT + APC therapy. Fifty-three patients who received APC therapy in our previous study were included as the control group. Following propensity score matching (PSM), we selected 54 patients (27 pairs). Baseline characteristics did not differ between these two groups before or after PSM (**Table 1**). The irradiation time and energy density used for each patient are shown in **Table 2**.

**Table 1:** Baseline general characteristics of patients.

Variable	Before PSM			After PSM		
	PDT ± APC group n (%)	APC group n (%)	*P*	PDT ± APC group n (%)	APC group n (%)	*P*
Total	28	53		27	27	
Age (years)						
Range	42-76	38-78	0.206	42-76	41-77	0.149
Mean	59.3	58.0		58.7	56.5	
Sex						
Male	17 (60.7)	28 (52.8)	0.497	16 (59.3)	14 (51.9)	0.584
Female	11 (39.3)	25 (47.2)		11 (40.7)	13 (48.1)	
Primary tumor						
ADC	15 (53.6)	24 (45.3)	0.478	13 (48.1)	13 (48.1)	1.000
SCC	13 (46.4)	29 (54.7)		14 (51.9)	14 (51.9)	
Tumor stage						
IVa	9 (32.1)	16 (30.2)	0.856	9 (33.3)	8 (29.6)	0.770
IVb	19 (67.9)	37 (69.8)		18 (66.7)	19 (70.4)	
ECOG performance status						
1	17 (75.0)	24 (45.3)	0.186	16 (59.3)	14 (51.9)	0.584
2	11 (25.0)	29 (54.7)		11 (40.7)	13 (48.1)	
Previous intrapleural treatment						
Yes	5 (17.9)	15 (28.3)	0.300	5 (18.5)	7 (25.9)	0.513
No	23 (82.1)	38 (71.7)		22 (81.5)	20 (74.1)	
Previous lines of systemic therapy						
0	8 (28.6)	6 (11.3)	0.055	7 (25.9)	5 (18.5)	0.407
1	16 (57.1)	29 (54.7)		16 (59.3)	14 (51.9)	
≥2	4 (14.3)	18 (34.0)		4 (14.8)	8 (29.6)	
Amount of MPE						
Medium	12 (42.9)	16 (30.2)	0.254	11 (40.7)	11 (40.7)	1.000
Large	16 (57.1)	37 (69.8)		16 (59.3)	16 (59.3)	

ADC, adenocarcinoma; SCC, squamous cell carcinoma; SCLC, small cell lung cancer; ECOG, Eastern Cooperative Oncology Group.

**Table 2:** Treatments and response for each patient.

Patient no.	Major lesions type	Intrapleural treatment	Irradiation time (min)	Energy density (J/cm^2^)	Response at 6 weeks	Response at 12 weeks
1	Plaque	PDT	8	384	PR	CR
2	Nodular	PDT+APC	10	480	NR	NR
3	Mass	PDT+APC	12	576	CR	CR
4	Plaque	PDT	8	384	PR	PR
5	Plaque	PDT	8	384	NR	PR
6	Nodular	PDT+APC	10	480	CR	CR
7	Mass	PDT+APC	12	576	PR	CR
8	Nodular	PDT+APC	10	480	CR	CR
9	Mass	PDT+APC	12	576	NR	NR
10	Plaque	PDT	8	384	CR	CR
11	Nodular	PDT+APC	10	480	CR	CR
12	Nodular	PDT+APC	10	480	PR	PR
13	Plaque	PDT	8	384	CR	CR
14	Nodular	PDT+APC	10	480	CR	CR
15	Mass	PDT+APC	12	576	PR	CR
16	Nodular	PDT+APC	12	576	NR	PR
17	Nodular	PDT+APC	12	576	PR	PR
18	Nodular	PDT+APC	12	576	CR	CR
19	Plaque	PDT	8	384	CR	CR
20	Mass	PDT+APC	12	576	CR	CR
21	Plaque	PDT	8	384	PR	PR
22	Nodular	PDT+APC	10	480	CR	CR
23	Mass	PDT+APC	12	576	CR	CR
24	Nodular	PDT+APC	10	480	NR	NR
25	Plaque	PDT	8	384	CR	CR
26	Nodular	PDT+APC	10	480	CR	CR
27	Nodular	PDT+APC	12	576	CR	CR
28	Nodular	PDT+APC	12	576	PR	PR

### Responses

**Figure 1:**
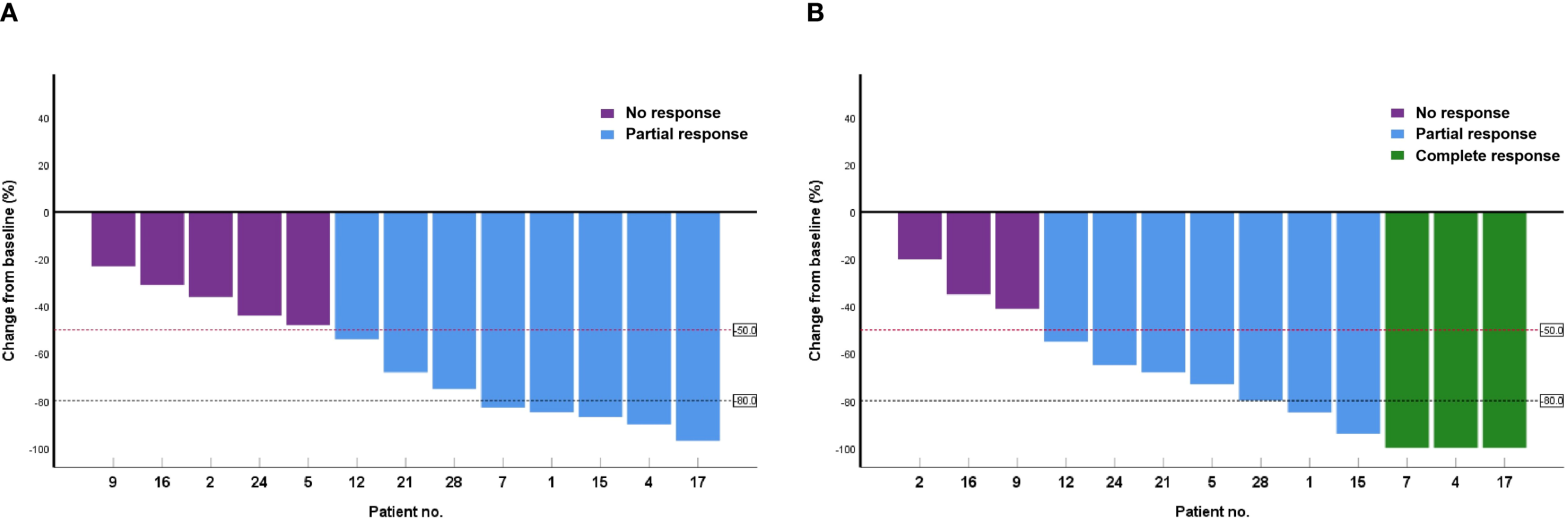
Waterfall plot of changes in malignant pleural effusion from baseline at weeks 6 **(A)** and 12 **(B)** in 13 patients.

At 6 and 12 weeks after treatment, the overall response rates (ORRs) in the observation group were 82.1% (15 cases of CR and 8 cases of PR) and 89.3%, respectively, with no significant difference from the APC group after PSM (*P* = 0.924 and 0.178, respectively). During the 6-week period between these timepoints, CR was maintained in 15 patients, PR improved to CR in 3 patients, NR improved to PR in 2 patients, and no patient in the PDT group developed disease progression. **Figure 1** is a waterfall plot of changes in MPE from baseline at weeks 6 and 12 for 13 patients (excluding the 15 patients with CR). In the control APC group, PR improved to CR in two patients and progression was detected in four patients (PR to NR in one case and CR to PR in three cases), resulting in the reduction of the ORR from 85.2% to 81.5% between 6 and 12 weeks (**Table 3**).

**Table 3:** Treatment response.

	Response at 6 weeks				Response at 12 weeks			
	Total	Matched comparison			Total	Matched comparison		
	PDT group n=28, (%)	PDT group n=27, (%)	APC group n=27, (%)	*P_1_* value	PDT group n=28, (%)	PDT group n=27, (%)	APC group n=27, (%)	*P*_2_ value
Complete response	15 (53.6)	15 (55.6)	14 (51.9)	0.924	18 (64.3)	18 (66.7)	13 (48.2)	0.178
Partial response	8 (28.5)	7 (25.9)	9 (33.3)		7 (25.0)	6 (22.2)	9 (33.3)	
No reponse	5 (17.9)	5 (18.5)	4 (14.8)		3 (10.7)	3 (11.1)	5 (18.5)	
Overall response	23 (82.1)	22 (81.5)	23 (85.2)		25 (89.3)	24 (88.9)	22 (81.5)	

### TTPs

**Figure 2:**
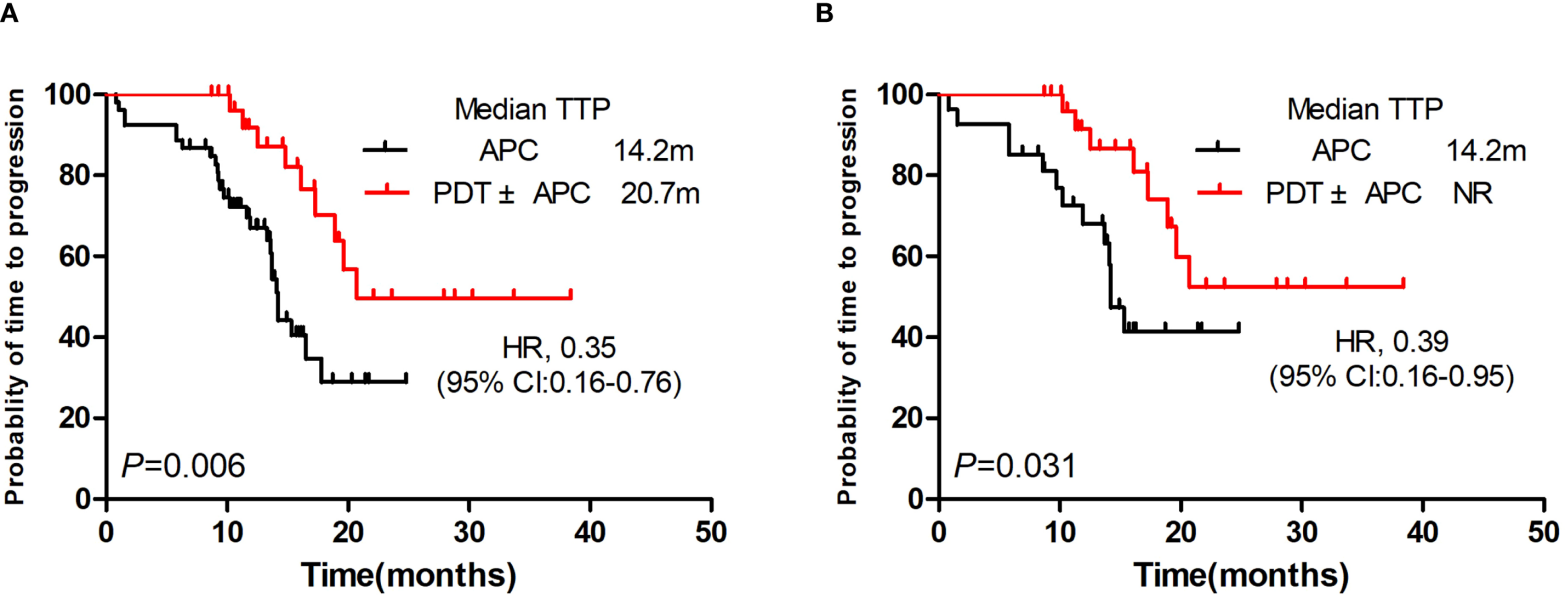
Kaplan–Meier curve of time to progression. Photodynamic therapy (PDT) ± argon plasma coagulation (APC) vs. APC before **(A)** and after **(B)** propensity score matching.

TTPs were significantly longer in the PDT ± APC group than in the APC group (median, 20.7 vs. 14.2 months; *P* = 0.006; HR = 0.35; **Figure 2A**). This difference persisted after PSM (*P* = 0.031, HR = 0.39). The median TTP had not yet been reached in the PDT ± APC group (**Figure 2B**).

### Subgroup findings

**Figure 3:**
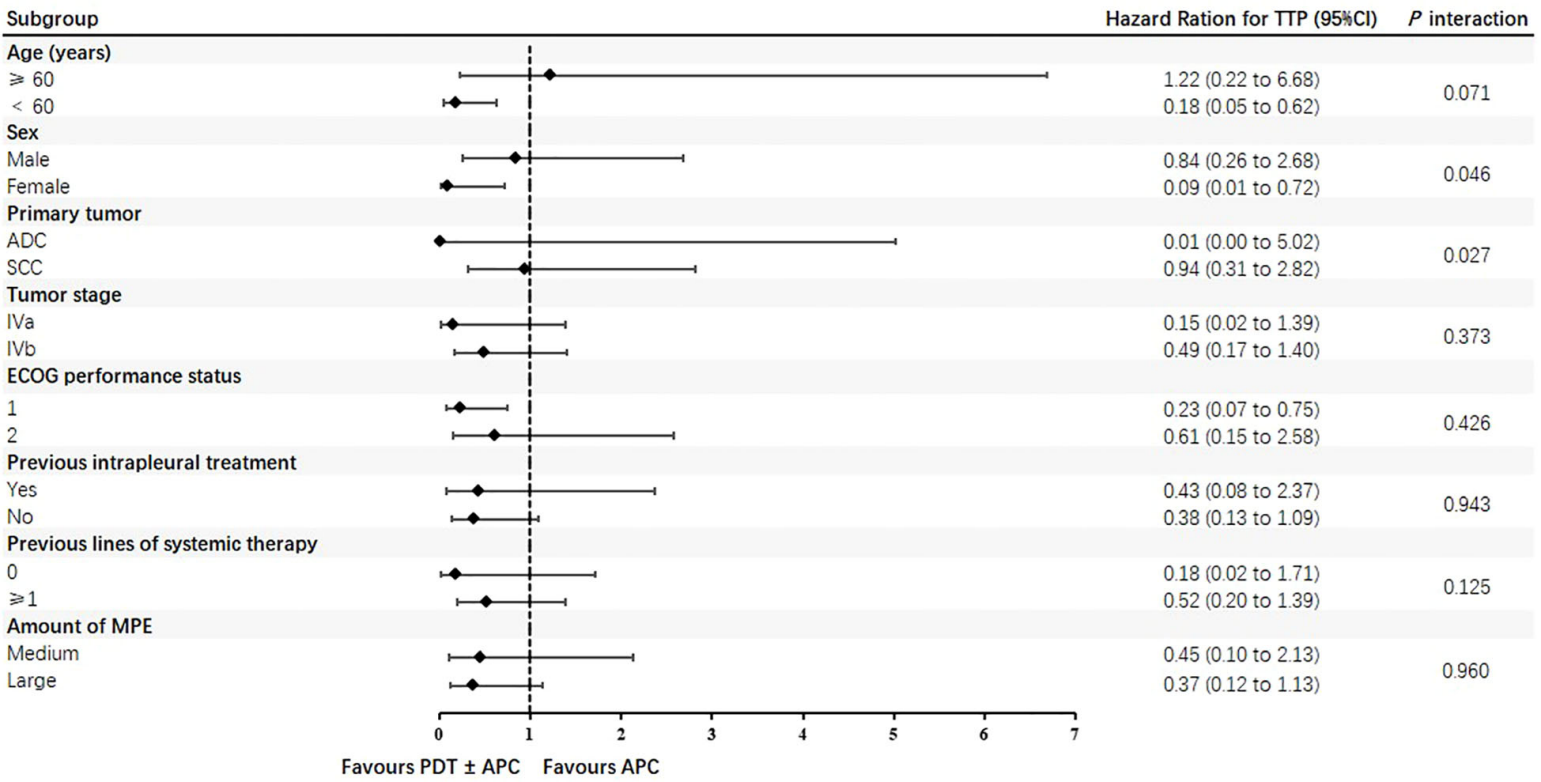
Forest plots of time to progression in photodynamic therapy (PDT) ± argon plasma coagulation (APC) and APC subgroups.

Subgroup analyses by major lesion type, intrapleural treatment, and energy density were performed for the TTP and ORR. The TTP was extended in patients with nodular pleural lesions, those who received PDT + APC therapy, and those treated with a 576-J/cm^2^ energy density, but not significantly so. Subgroup findings for the TTP in the PDT ± APC and APC groups are presented as forest plots in **Figure 3**. HRs for the TTP were <1.0 for almost all subgroups. In addition, the upper limits of 95% CIs were <1.0 for the age < 60 years, female sex, and ECOG score < 1 subgroups. Subgroup ORRs at weeks 6 and 12 are provided in **Table 4**.

**Table 4:** Subgroup analysis of time to progression and overall response.

	N	Time to Progression			Overall Response, n (%)	
		Mean, month	95% CI	*P*	6 weeks	12 weeks
Major lesions type						
Plaque	8	24.0	16.93-31.15	0.819	7 (87.5)	8 (100)
Nodular	14	27.2	19.93-34.56		11 (78.6)	12 (85.7)
Mass	6	25.6	20.24-31.01		5 (83.3)	5 (83.3)
Intrapleural treatment						
PDT	8	24.0	16.93-31.15	0.783	7 (87.5)	8 (100)
PDT+APC	20	28.3	21.93-34.59		16 (80.0)	17 (85.0)
Energy density (J/cm^2^)						
384	8	24.0	16.93-31.15	0.938	7 (87.5)	8 (100)
480	9	23.0	17.39-28.65		7 (77.8)	7 (77.8)
576	11	30.3	22.63-38.04		9 (81.8)	10 (90.9)

### One-year survival rate

One-year survival rates were 75% (21/28) in the PDT ± APC group and 66% (35/53) in the control APC group. For comparison, reported 1-year survival rates for other intrapleural treatments (talc pleurodesis, cytoreductive surgery, and hyperthermic intrathoracic chemotherapy) are provided in **Table 5**.

**Table 5:** Comparison with other treatments for patients with malignant pleural effusion.

First author[Ref.]	Intrapleural treatment	No. of patients	Type of malignancy	1-year survival rate
Davies (12)	Talc pleurodesis	54	Lung, Breast, Mesothelioma, Others	13.0% (7/54)
Thomas (13)	Talc pleurodesis	71	Lung, Breast, Mesothelioma, Others	28.2% (20/71)
Migliore (29)	Cytoreductive surgery and heperthermic intrathoracic chemotherapy	21	Lung (Adenocarcinoma, Adeno-squamous)	61.9% (13/21)
Hassan M (30)	Cytoreductive surgery and heperthermic intrathoracic chemotherapy	31	Lung, Ovarian, Sarcoma, Others	77.0%
Mai (8) (Our previous study)	Argon plasma coagulation (APC) therapy	53	Lung (Adenocarcinoma, Squamous-cell carcinoma)	66.0% (35/53)
Mai (Our present series)	**Photodynamic therapy ± APC**	**28**	**Lung (Adenocarcinoma, Squamous-cell carcinoma)**	**75.0% (21/28)**

The boldfaced results are derived from our current study.

### Safety

The toxicities related to treatment observed in this study were notably minor. No severe complication, such as pleural fistula or acute respiratory distress syndrome resulting in death occurred. Capillary leak syndrome occurred in one patient in poor condition (serum albumin concentration < 30 g/L and electrolyte disturbances) with a large tumor burden. This patient experienced dyspnea and sudden drops in blood pressure and oxygen saturation at 30 hours postoperatively. Wet rales were detected in both lungs. After timely tracheal intubation, mechanical ventilation, and the administration of a glucocorticoid, diuretics, and other comprehensive symptomatic supportive treatment, the patient’s condition was effectively controlled.

## Discussion

In this exploratory pilot study performed to evaluate the clinical efficacy of PDT with and without APC for MPE with pleural disease, the ORR at 6 weeks after treatment was satisfactory, in line with our previous findings for APC therapy (8). This response was found to be durable, with a trend toward a higher ORR at week 12 that was not observed in the APC group. Moreover, the 1-year survival rate was up to 75%, even though these patients had not received targeted therapy or immunotherapy and had received second or more systemic treatment lines, which tend to be associated with worse prognoses (12, 13). The median TTP for the observational group in this study far exceeds that representing our institutional experience with APC therapy without PDT for MPE. Subgroup analyses suggested that PDT + APC and the use of 576 J/cm^2^ energy density tend to prolong the TTP, although the results were not significant. These findings suggest that PDT with or without APC is more effective and offers more potential advantages than does APC therapy alone.

Theoretical factors underpin the potential advantages of PDT in the treatment of malignant disease. The greater retention of photosensitizers in cancer cells than in normal cells enables tumor targeting with the preservation of normal organ function (14). In addition, the visible light used to activate the photosensitizers has a shallow tissue-penetration depth of only several millimeters and covers a wide radiation range, resulting in the killing of superficial cells while preserving underlying tissues (15). Thus, PDT covers lesions in the visceral pleura and residual invisible cancer lesions, and is suitable for the treatment of superficial pleural dissemination with no fear of pleural fistula development. Moreover, vascular endothelial growth factor (VEGF) induces vessel hyperpermeability and is reportedly associated with MPE development (16–18). PDT may not only reduce the pleural tumor burden by inducing the apoptosis of tumor cells, but also inhibit the proliferation of vascular endothelial cells by downregulating VEGF expression and damaging the tumor vasculature (19–23). PDT is also a potential component of combination pleural tumor therapy. Pleural metastasis can appear as multiple masses of various sizes or nodular lesions in addition to superficial spreading. For these lesions, PDT alone is not adequate due to its shallow penetration depth. We hypothesized that the use of APC therapy to ablate gross tumors as much as possible, followed by PDT to irradiate residual tumors and surrounding areas, would improve local control and potentially overall survival. Thermal ablation, as well as PDT, may activate a systemic or localized antitumor immune response (24–26). Thus, the use of PDT with APC and systemic antitumor therapy may synergistically enhance curative effects without causing overlapping adverse reactions.

In a phase-II trial of the use of pleural PDT with surgery in the treatment of NSCLC with pleural spread, a 6-month localized disease control rate of 73.3% and a median overall survival time of 21.7 months were achieved; the median survival time for similar patients treated with the nonoperative standard was 6–9 months (27, 28). The treatment strategy employed in that trial was the surgical resection of gross disease and intraoperative PDT for residual microscopic disease. Alternative therapies for pleural metastases reported in the literature, such as cytoreductive surgery combined with hyperthermic intrathoracic chemotherapy (HITHOC), require debulking via thoracotomy. Migliore et al. and Hassan M et al. reported 1-year survival rates of 61.9% and 77%, respectively, figures that are broadly in line with the 75% observed in our study (29, 30). It must be emphasized, however, that these data are not derived from head-to-head comparisons; any direct discussion of survival benefit therefore warrants extreme caution. Although the inherent limitations of medical thoracoscopy preclude complete macroscopic tumor removal, we nonetheless achieved excellent local control of MPE and an encouraging 1-year survival rate.

Although the response observed in this study was durable, three patients showed NR at week 12. Serious pleural hypertrophy, pleural adhesions, and atelectasis for >2 months were observed in these patients and may be predictors of poor PDT efficacy. Systemic medical therapy is the cornerstone for the pleural metastasis of lung cancer. We did not include patients who had received targeted therapy or immunotherapy, which have good anti-NSCLC effects, in this study. However, it would be a mistake to not mention the need for the continued improvement of systemic anti-tumor treatment, as 64.3% (18/28) of our patients in whom CR was achieved had well-controlled primary and metastatic lesions.

### Limitations

Although the results of this study are promising, medical thoracoscopic PDT is still in the clinical exploration stage. Further studies are needed to optimize the laser irradiation dose and operative process, ultimately improving treatment efficacy. Additionally, this study had a single-arm design and was performed with a small sample and comparison with APC therapy data from our previous study, which inevitably introduced bias. Thus, prospective multicenter randomized controlled trials are needed to provide more robust evidence supporting the use of PDT in this context.

### Conclusions

With proper patient selection, medical thoracoscopic PDT ± APC therapy seems to be feasible for the treatment of pleural spread in patients with NSCLC and malignant effusion. This treatment significantly improved TTP relative to APC alone and provided durable local effusion control. PDT and APC therapy can complement each other, especially for the treatment of nodular or mass-like lesions. This combined multimodal therapy is safe and controllable. The response, TTP, and survival results from this study are encouraging, and further multicenter investigations are warranted.

## Data Availability

The original contributions presented in the study are included in the article/supplementary material. Further inquiries can be directed to the corresponding authors.
